# Winter Wisdom

**Published:** 1874-01

**Authors:** 


### WINTER WISDOM.

KEEP your feet always dry and warm, first, by keeping them clean. In slushy, muddy weather, it is better to wear rubber shoes, if for not longer than two or three hours; but for those who are on their feet all day, standing, or walking in melting snow, or on damp ground, it is better to have thick soled shoes, the bottoms of which have been saturated in common grease, or painted several times with kerosene especially at the joinings with the upper leather.

If a shoe is "tight" to the feet, it prevents the circulation and hinders the generation of natural warmth, hence in winter the shoes should be moderately large. In having a new pair made to measure, put on two pair of woolen stockings, then the new shoe, when worn over but one pair, will be most comfortably easy.

If the feet are cold while riding in vehicles keep them off the floor, and remember that a newspaper will keep the feet warmer than a thick, tight leather boot.

Some persons can keep their feet warmer in cotton than woolen stockings, while some do better to have two thin pair than one of a thicker material.

Neither silk, cotton nor linen are safe or suitable materials for wear next the skin, especially in winter, or from October to June; it is positively dangerous to do so on the part of the young, the old, the frail and the feeble, because we are not warmed by what we wear. The warmth which keeps us alive comes from within, hence those materials are best for warmth which are known to keep in and retain that heat which is generated within the body—generated under the skin and not outside of it. Such bodies are known among scientific men as "non-conductors;" that is, they do not convey the heat away, but rather absorb and retain it. Woolen fabrics are the best non-conductors known, hence all sleep under blankets in winter. Irish linen is notably a conductor, silk is less so, while cotton stands between them and woolen.

The best material for common use, to be worn next the skin, is the old-fashioned flannel shirt; white is the most acceptable color, but it becomes too close and leathery by frequent washing. Colored woolen flannel shirts do not "shrink" up much, and are, therefore, better on that account. White flannels can be washed without much shrinkage by dipping them in boiling soap-suds, and, after remaining for a while, do not wring them dry, but hang them on the line, and let them drip dry.

Old persons, and those who are of frail constitutions, should wear woolen shirts and drawers also; for such, and for those, too, who ride on horseback in very cold weather, knitted woolen shirts and drawers are best, they being much thicker than flannel.

Many persons have brought on serious, dangerous, and even fatal sickness, by going to the front door with visitors, holding on to the door knob while the door is partly open, in taking "last words."

In going out into the cold air from a warm room, many leave the buttoning of the coat and drawing on the gloves to be done after they are outside the door, and sometimes get thoroughly chilled before it is accomplished, especially the fingers; hence adjust all these things in the house, and on emerging into the open air keep the mouth resolutely closed for the first five or ten minutes, to warm the air before it gets to the lungs.

